# Determinant Factors in Personal Decision-Making to Adopt COVID-19 Prevention Measures in Chile

**DOI:** 10.3390/ijerph18158131

**Published:** 2021-07-31

**Authors:** Karina Fernanda Gonzalez, Maria Teresa Bull, Sebastian Muñoz-Herrera, Luis Felipe Robledo

**Affiliations:** 1 Department of Industrial Engineering, Universidad Catolica de la Santisima Concepción, Concepcion 4090541, Chile; kgonzalez@ing.ucsc.cl (K.F.G.); sebastian.herrera@ucsc.cl (S.M.-H.); 2 Observatory of Disaster Management (OGD), Universidad Católica de la Santísima Concepción, Concepción 4090541, Chile; luis.robledo@unab.cl; 3 Engineering Science Department, Universidad Andres Bello, Santiago 7500971, Chile

**Keywords:** COVID-19, risk homeostasis, risk perception, Protective Action Decision Model, prevention measures adoption, exploratory study

## Abstract

The pandemic has challenged countries to develop stringent measures to reduce infections and keep the population healthy. However, the greatest challenge is understanding the process of adopting self-care measures by individuals in different countries. In this research, we sought to understand the behavior of individuals who take self-protective action. We selected the risk homeostasis approach to identify relevant variables associated with the risk of contagion and the Protective Action Decision Model to understand protective decision-making in the pandemic. Subsequently, we conducted an exploratory survey to identify whether the same factors, as indicated in the literature, impact Chile’s adoption of prevention measures. The variables gender, age, and trust in authority behave similarly to those found in the literature. However, socioeconomic level, education, and media do not impact the protection behaviors adopted to avoid contagion. Furthermore, the application of the Protective Action Decision Model is adequate to understand the protective measures in the case of a pandemic. Finally, women have a higher risk perception and adopt more protective measures, and in contrast, young people between 18 and 30 years of age are the least concerned about COVID-19 infection.

## 1. Introduction

The World Health Organization (WHO) alerted the population to COVID-19 in 2019. Despite government efforts, the virus has caused more than 21.8 million infections and more than 570 thousand deaths in South America. Chile has presented one of the highest fatality rates (deaths compared to confirmed), reaching 2.28% in April 2021. The pandemic has changed people’s daily lives, mainly due to changes in routine and habits, including confinement due to quarantine (preventive or mandatory isolation to reduce the risk of spreading COVID-19), social distancing, the use of a mask, changes in the income of each household, among others [1]. However, prevention measures such as isolation cause a change in psychosocial well-being, generating an impact on people’s mental health [2].

Today, the challenge presented in disaster management by the pandemic is that it is a long-run disaster that changes people’s lives, in many cases, more than the changes resulting from other disasters such as tsunamis, earthquakes, and volcanic eruptions. Therefore, in the pandemic case, individuals must make decisions regarding personal protection, especially when people’s behavior in not adopting self-prevention measures is indicated as one of the causes leading to an increase the number of infected people.

In the literature, we can observe that different factors affect this decision-making, especially those that depend on each individual’s perception, called organization perception and interpretation of sensations [3]. This perception makes it possible to generate awareness of all events abroad, including costs and benefits. Thus, the theory of risk in decision-making arises, in which decision-making is analyzed with uncertainty in the results so that the consequences are not defined in advance but are subject to change [4].

Based on the literature, we can identify the factors that can influence people’s decision-making in cases of a natural hazard (earthquakes, tsunamis, and hurricanes), but we do not know the factors in a pandemic case, nor how people perceive and accept the risk of the new disease. Therefore, this research seeks to identify and characterize the factors that impact decision-making regarding prevention measures against COVID-19 in the literature and establish if the same variables influence people’s decision-making to adopt protection measures in Chile.

## 2. Decision-Making for Individuals at Risk of Disaster

### 2.1. Approach for People’s Response and Behavior

Different approaches have attempted to model decision-making processes on people’s responses to environmental hazards recently. The Protective Action Decision Model (PADM) proposed by Lindell and Perry (2004) and later improved by the same authors in 2011 [5] is one example. The advantage of this model is that it identifies different aspects that affect people’s protection behavior in facing a disaster from a theoretical perspective. This conceptual framework proposes three core perceptions: threat perceptions, protective action perceptions, and stakeholder perceptions. These perceptions are based on environmental and social cues and the information associated with warning messages. After the perceptions are formed, the individual can decide the protective action and generate a behavioral response. However, this response can be influenced by situational facilitators or situational impediments present in their habitat.

Researcher Gerald Wilde in 1982 proposed the risk homeostasis theory, where decision-making in uncertain environments depends on the risks that individuals face and the psychological conditions of the individuals themselves concerning that type of risk. The risk homeostasis theory postulates that people always generate a comparison of the amount of risk they perceive with their objective or tolerated level of risk and adjust their behavior to eliminate any discrepancy between the two [6]. In other words, individuals faced with a risky situation try to balance their attitude according to what they believe to be the risk and not according to the actual risk. Therefore, this individual risk thermostat would explain the different behaviors of individuals facing the same risk situation.

Likewise, this means that the individual accepts a certain level of subjectively estimated risk, both for his health, safety, and other assets, in exchange for the benefits he receives from it. Wilde takes this concept further by indicating that individuals have a level of injury risk that they are willing to accept. So, according to Wilde [6], the decision and the level of risk tolerated depends on the following four factors:

1. The expected benefits of risky behavior.
2. The expected costs of risky behavior.
3. The expected benefits of safe behavior.
4. The expected costs of safe behavior.

Wilde [6] (p. 1) also indicates that the level of risk tolerated by the individual will be riskier as factors 1 and 4 are higher and factors 2 and 3 are lower.

Considering the approach proposed by Wilde, risk perception has a variation between people. Additionally, he refers to the fact that each person performs a cost and benefit comparison of each action under a dangerous situation [6] (p. 2). Thus, based on the theory of risk homeostasis and the influence of perception for people in risky situations, several factors have been identified as influencing decision-making, among them variables related to knowledge of the disease, personal and emotional experience, the sociocultural paradigm, gender, and age [7], as well as psychosocial and geographical characteristics [8,9].

### 2.2. The COVID-19 Impact

At the end of 2019, specifically on December 31, a disease associated with coronaviruses was made known by the World Health Organization [10]. This disease called COVID-19 is a highly infectious disease caused by the new coronavirus known as SARS-CoV-2. This disease came to cause an epidemic of acute respiratory syndrome. The infected people display the following common symptoms of fever, dry cough, and fatigue. Additionally, less common symptoms reported include aches and pains, nasal congestion, headache, conjunctivitis, sore throat, diarrhea, loss of taste or smell, skin rashes, or color changes on the fingers or toes [11]. These symptoms allow an alarming spread through droplets emitted by coughing, sneezing, or talking with an infected person, so on 11 March 2020, the World Health Organization declared the COVID-19 situation had changed from epidemic to pandemic [2], affecting several countries since it originated in Wuhan, China [9,11].

People’s social behavior has changed due to COVID-19. For example, the distance between two individuals must be at least one meter and they must wear a mask daily to go out home to avoid being infected. These changes came to disrupt everyone’s daily life and the high rate of spread and mortality, adding another health condition, have caused collective anxiety [12,13]. Additionally, the World Health Organization indicates that people older than sixty and people with underlying medical conditions—such as high blood pressure, heart or lung problems, diabetes, obesity, or cancer—are more vulnerable to this disease [10].

In Chile, the first case of COVID-19 occurred on 3 March 2020 [14], and the first case of death from COVID-19 was on 21 March [15]. This country implemented a plan to confront the pandemic called the Step-by-Step plan, which is a gradual strategy to confront the pandemic according to the sanitary situation of each town. It consists of four scenarios or gradual steps, ranging from lockdown (first step) to initial opening (fourth step), with specific restrictions and obligations. From one phase to another, progress or regression is subject to epidemiological indicators, health care networks, and traceability.

Therefore, this pandemic unleashed many changes in the daily life of every inhabitant in Chile; for example, the suspension of classes in educational institutions, economic changes and layoffs affecting the economic situation in many households, changes in work modalities, and limitations in the mobility of the inhabitants due to sanitary lockdown of different cities, and the requirement of different official documents to move from one city to other. Additionally, to decrease the number of people infected, the Health Ministry promotes the use of masks, preventive isolation, frequent hand washing, home ventilation, distancing between people and, finally, getting daily information from reliable sources on the progress of the disease [11]. Unfortunately, all these changes in daily life have caused mental health problems, evidenced as post-traumatic stress, confusion, and anger [16]. According to Villagrán (2020) [16], mental health problems can affect each person’s decision-making process and then the actions related to the self-care of everyone.

## 3. Modeling Decision-Making to Face COVID-19 Pandemic

Commonly, choosing how to face a hazard, selecting between following the policies previously indicated by authorities or behaving according to one’s own policy, is not an easy choice because it is affected by several aspects. The risk homeostasis approach uses the risk perception and the level of risk accepted by the individual to explain the decision-making process. Although Wilde considers that people change their behavior based on factors within this health and safety approach, individuals will not change their behavior unless some measures motivate people to adjust the amount of risk they are willing to accept [17]. Lindell and Perry [5], in their Protective Action Decision Model (PADM), also mentioned some aspects that had an impact in threat’s perception, protective action perceptions, stakeholder perceptions, and the auto protection needed to deal with a hazard.

Changing behavior and motivations are essential when analyzing the lack of self-care measures taken by the population in facing the COVID-19 pandemic [18]. In addition, we know from past pandemics that the success of policies to curb the rapid transmission of a highly infectious disease depends, in part, on the public having an accurate perception of personal and social risk factors [19]. Currently, each person is facing new and unexpected situations considering their personalities and vulnerabilities. Although there are different vulnerabilities and personalities, the literature identifies some critical factors that help people perceive the disease’s severity in their communities.

### 3.1. Leading Factors Affecting Decision-Making

**Figure 1:**
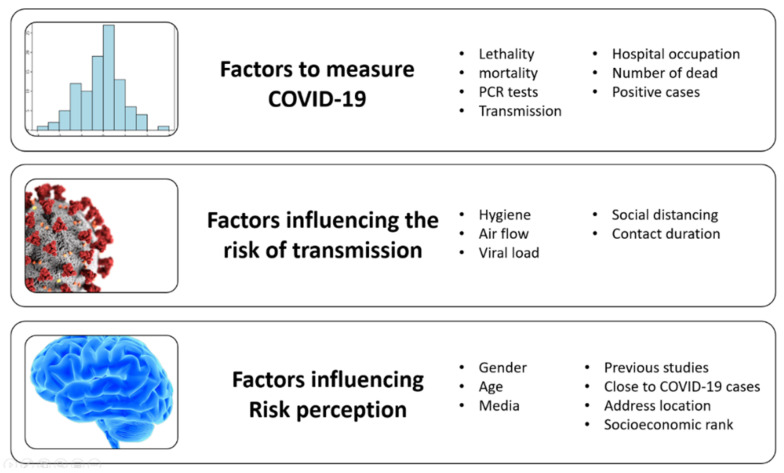
Concepts associated with COVID-19.

COVID-19 forced the community to become familiar with different new terms that the literature indicates are relevant to understand the epidemiological situation in each country. All these new topics also impact the perception of the risk in the community. In the literature, we find several concepts that we classify as factors to measure the severity of the COVID-19 in the country, factors that measure the risk of transmission, and characteristics of the population that impact the level of risk perceived by the community, Figure 1 summarizes the factors identified in the literature.

### 3.2. Modeling Decision-Making

**Figure 2:**
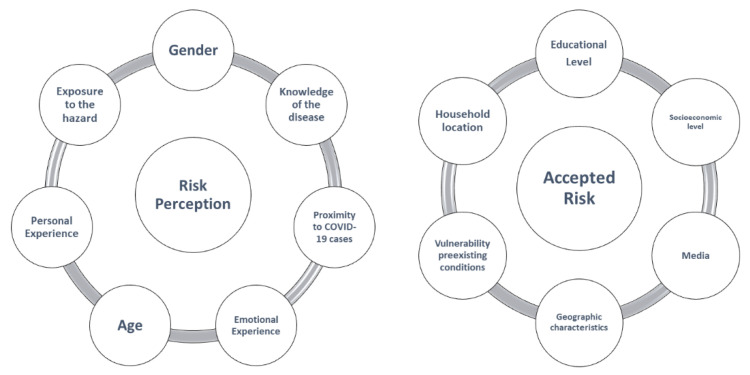
Factors influencing people’s perception of risk.

In this research, we adopted the risk homeostasis approach to classifying the different factors found in the literature concerning risk. This approach allowed us to identify the factors in Figure 2 that generate the perception of risk and the conditions of people that define the level of accepted risk.

**Figure 3:**
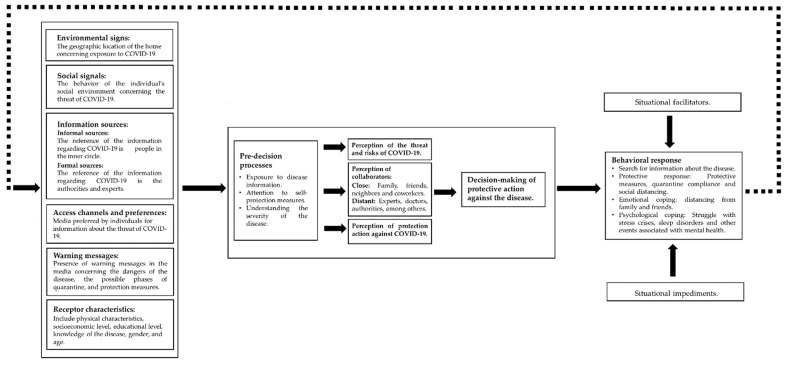
Application of the model to the situation of protective decision-making in the case of COVID-19.

After identifying the factors that impact risk perception, we seek to apply the context of protective action decision-making proposed by Lindell and Perry [5] to the specific case of the pandemic. Thus, we model the decision-making process for the COVID-19 pandemic to understand the decision-making process of the population and seek the reasons for the low adoption of protection measures promoted by the government through formal channels. Figure 3 shows the application of the model to the situation of protective decision-making in the case of COVID-19.

In general terms, the Protective Action Decision Model postulated by Lindell & Perry in 2012 adapts and works in a very appropriate way with the data obtained based on the factors identified that affect decision-making when facing a risky situation, such as the pandemic, in the literature (see Figure 3). However, it has some shortcomings when discussing a disease since this pandemic is not as common a disaster as an environmental catastrophe (earthquake, tsunami, hurricane, among others). Socio-natural disasters generally affect the population over shorter periods, unlike the pandemic, in which it is unknown how long it will take for the population to return to the normality experienced before the appearance of COVID-19. This difference made the population drop some protection measures through the duration of the pandemic due to the tiredness associated with the restrictions on social behavior. Then, in case of a pandemic, the situational facilitators and the situational impediments increase the importance of adopting protective measures in the long run and increase the importance of the psychological aspect in modeling the behavior to adopt protection actions. As shown in Figure 3, we added psychological coping, since this feature is of great importance on some occasions, to generate effective protective action in the individual.

## 4. Material and Methods

The main objective of this study was to identify and evaluate relevant characteristics and factors when taking a self-protection action against risk, and additionally, to identify the differences in people’s perceptions and contrast them with those mentioned in the literature. For this purpose, an exploratory survey has been carried out.

The survey was applied in December, ten months after the first case in Chile. At that moment, most of the counties were on the first step according to the Step-by-Step plan, which meant lockdown in most of the towns in the central south of Chile, where the survey was done online (see the survey in Appendix A). The survey included three sections. The first section tried to collect individuals’ personal information to generate an environment of trust for the reader and, in turn, verify if there were differences between the gender of the individuals, their ages, and their dwelling type. The second section had questions directly related to risk perception, with questions related to the degree of concern about the disease. In the same way, it sought to know if the respondent had been close to infected people and their perception of their own mental health. Then, in the final section, questions were asked related to the different scenarios or factors that can influence a change in risk perception, thus generating behavior modification in self-protection actions. In this section, we requested information related to trust in government authorities or the media, level of participation in events (unavoidable or not) outside the home, economic situation, and protection mechanisms.

### 4.1. Data Collection Procedures

For this study, we developed an anonymous online survey designed to collect detailed information from people regarding their attitudes to COVID-19, their actions to protect themselves from COVID-19, and housing conditions. The 26-item survey was divided into three sections: personal background, perceptions, and factors influencing the perceptions. Subjects were recruited in two ways: (1) the electronic link for this survey was via broadly distributed via WhatsApp message; (2) through the networks of those who received the WhatsApp message, who were encouraged to share the survey link with individuals older than 18 years. All potential participants were given the option to choose not to participate and leave the survey at any moment. This recruitment of a sample was done between 23 December 2020 and 17 January 2021, resulting in 186 responses. The authors estimate that the invitation was sent to 400 people, and the rate of answers was 46.5%. The participants responded to the complete survey, and we considered all the 186 responses as our sample for our exploratory research. This study was conducted according to the guidelines of the Declaration of Helsinki, and approved by the Ethics, Bioethics, and Biosafety Committee of Universidad Catolica de la Santisima Concepcion, Concepcion, Chile (protocol code 21/2021).

### 4.2. Participants Description

The survey was answered by 186 people from different parts of Chile, of whom 128 were women, and 58 were men. The sample was mainly composed of 88.17% people from the Biobío Region, the third most populated region in Chile, and 6.45% people from the Metropolitan Region, where the capital of Chile is located. In terms of age, 39.25% of the respondents were in the 18–30 years range, 32.26% of the respondents were 31–45 years old, 23.12% were 46–60 years old, and only 5.38% were older than 60 years.

### 4.3. Instrument

The survey design was based on obtaining information oriented to measure the factors identified in the literature that affect the protective decision-making process under the threat of COVID-19. Three sections were generated. The first was to obtain classification information from respondents, the second section aimed to seek information on risk perception, and the third to measure the factors affecting perception. For each of the questions, respondents were asked to click on at least one of the alternatives indicated to facilitate their response. The complete survey is in Appendix A.

### 4.4. Statistical Analysis

We conducted descriptive statistical analysis for most of the variables and then analyzed the relationship between the individuals’ protection actions and features of the population. To validate the difference between men and women, we used the chi-square test for proportions.

## 5. Results of an Exploratory Study of the Factors for Self-Protection in Chile

According to the factors highlighted in the literature, the main findings of this research are the following:

### 5.1. Gender

**Figure 4:**
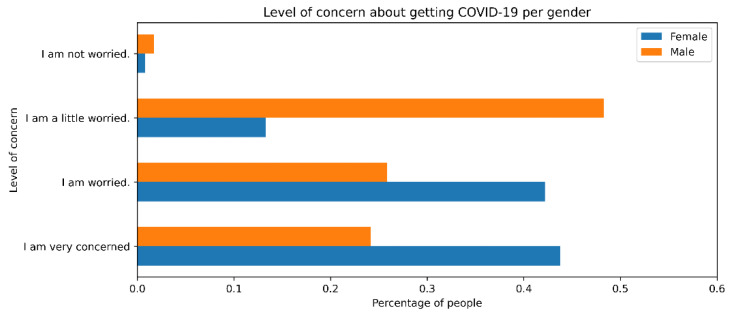
How worried are you about getting COVID-19 (by gender).

As shown in the literature [9,13], the survey results showed differences between genders. The perception of risk was higher in women (43.75%) than in men (24.14%) when asked about the degree of concern about the health emergency (see Figure 4). Likewise, women presented a greater sense of fear in the face of the pandemic, so they adapted more quickly to adopt self-protection measures. However, when asked how often they used any self-protection methods, the percentages were very similar. Thus, both genders were willing to use protective methods, although women were more concerned about COVID-19. In our statistical results, *p*-value < 0.01 for the chi-square test. Thus, we can conclude a significant relationship between gender and the perception of risk of the individual.

### 5.2. Age

The survey was designed to obtain information on three age groups: people between 18 and 30 years old (73 respondents), between 31 and 45 years old (60 respondents), and people over 45 years old (53 respondents). The results indicate that the concern about a COVID-19 infection is more significant for older ages. Thus, only 26.03% of the first group presented a high degree of concern, with an increasing trend of 38.33% and 52.8% for the other segments, respectively. The relationship between age and the person’s concern about the virus was significant according to the chi-square contingency test with a *p*-value = 0.003. Additionally, we validated this by analyzing the number of times they attended group events. There was a significant difference (*p*-value < 0.05) between the different age ranges, since the youngest respondents claimed to have attended events (78.1%), with this response decreasing as age increased (43.4% for the last group).

### 5.3. Socioeconomic Level

**Figure 5:**
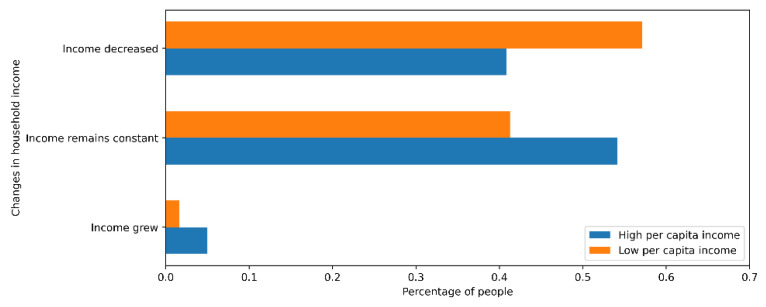
Changes in household income during the pandemic.

As expected, the pandemic has harmed Chilean families, especially those belonging to the three lowest quintiles, in which 57.1% of people have seen their income decrease, unlike the two highest quintiles, in which 40.8% had a reduction (see Figure 5).

On the other hand, there were no appreciable differences between the three lowest quintiles and the two highest in attendance at shopping centers or restaurants. Forty percent of those surveyed admitted to having visited this type of establishment in the past month. As a complement, the percentage of people really concerned about the pandemic was close for both segments, reaching 32% in the lowest quintiles and 40% in the highest quintiles.

### 5.4. Closeness to COVID-19 Cases

**Figure 6:**
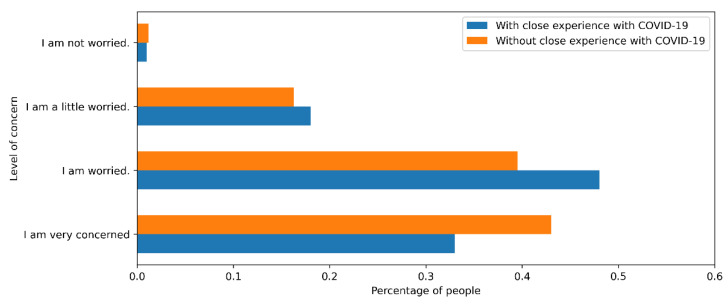
Risk perception based on closeness to COVID-19 cases.

Individuals who had close experiences with the virus tended to be more concerned about becoming infected and showed an increase in risk perception [9]. However, the survey shows that people who had not had any closeness to the disease were equally worried about the contagion as people who had experience of the virus (see Figure 6). We can explain this phenomenon by the severity of the symptoms experienced by people in their social circle. In subsequent investigations, we need to characterize the severity of nearby cases to verify this relationship. One hypothesis is that when such cases presented slight symptoms or were asymptomatic, people from their close circle usually showed a false sense of security, mistakenly thinking COVID-19 had mild effects, even though reality shows that symptoms vary for each individual [10].

### 5.5. Media

In the literary review, we observed that it is essential to consider that all the information media influence the change in people’s perception of risk [2], where the lack of perception of risk is due to the little or poor information to which one has access. The survey revealed that the media most used by individuals when informing themselves about the progress of COVID-19 were social networks and television, with 41% and 38% use, respectively: radio (15.4%) and newspapers (4.8%) received little attention. In a complementary way, we studied behavior according to age. Adults between 18 and 30 years old mostly preferred the use of social networks (91.8%), television (73.6%), and radio (13.7%). In adults between 31 and 45 years old, the percentages were 61.7%, 56.7%, and 30%, respectively. On the other hand, among adults over 45 years of age, the preferred information medium was television (75.5%), followed by social networks (69.8%) and radio (34%).

In addition, a loss of confidence in the information provided by the media stood out. In this context, only 26.34% of people had a high level of trust in the media, while 55.38% of people only partially trusted the information provided.

### 5.6. Perception of Mental Health

**Figure 7:**
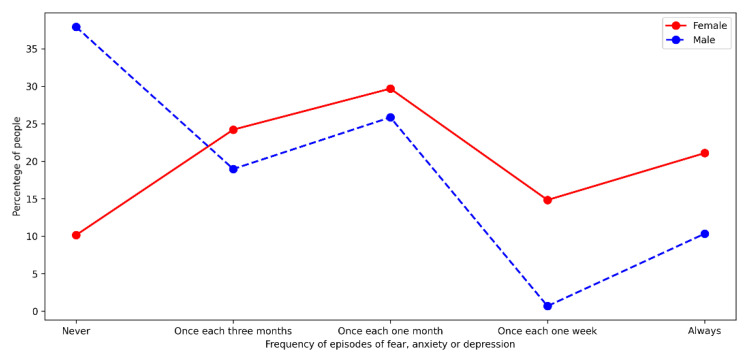
Mental health during pandemic.

Concerning perceptions of mental health, we consulted on the frequency with which people experienced fear, anxiety, or some symptom that they could attribute to a depressive state during the pandemic. We separated responses by gender and considered five categories of frequency of events associated with mental health. According to their perception, we classified respondents into four categories: never had a symptom, had symptoms all the time, and those who had them every week, every month, or every quarter. This categorization made it possible to analyze the effect of gender when respondents faced an exploratory consultation on mental health. Most male respondents (57%) declared not having experienced any of the symptoms described (see Figure 7).

On the other hand, only 10.16% of the female population declared the same. Additionally, male respondents rarely admitted experiencing episodes of this type since 82.76% indicated that their episodes with these symptoms were over periods greater than one month. On the other hand, in women, the percentage of people in each category was more homogeneous. The correlation between gender and mental health perception is significant (*p*-value < 0.001). In this context, we may interpret the results through one of these two approaches: (1) the female population has a greater awareness of their mental health compared to the male population; or (2) the male population identifies the symptoms but does not see them as a health problem, which causes a minimization of some episodes or symptoms.

### 5.7. Trust in Authorities

Another aspect that we analyzed was the confidence that people have about the information provided by the authorities about the pandemic. Around 23% of those surveyed indicated that they did not have any confidence in the information provided by the government. In addition, those who indicated they had little confidence represented a large proportion of respondents in each age segment, reaching 64% in adults between 18 and 30 years old, 40% in the range between 31 and 45 years old, and 56.6% in the oldest group. In this context, the relationship between age and trust in authorities is significant (*p*-value = 0.0118).

On the other hand, regardless of the media used, people had low trust in the authorities. In this context, 24.8% of the people who used social networks as their primary media stated that they did not trust the authorities, with lower percentages for those who preferred television (16.5%) and radio (4.35%).

### 5.8. Exposure to the Hazard

The percentage of people who declared having met with people outside their family circle was higher in younger people (91.78%) than other age groups; 73.3% of people between 31 and 45 years old indicated that they had spent time with people outside their family circle, and the figure was less in the oldest group (56.6%). Additionally, we asked whether exposure to the risk of contracting COVID-19 had occurred in a work, mobility, or leisure context. The results indicate that the youngest age group was the one that admitted to having had a greater exposure for leisure reasons (56.2%), either by attending events or eating out. This decreased to 35% and 22.6%, respectively, for the two oldest groups.

Regarding exposure for work reasons, the group between 31 and 45 years old was the one that had the most significant exposure: 55% of those surveyed admitted to having had contact with people outside their family circle due to work, while the percentage was lower for the youngest group (38.4%). Additionally, 58% of the group members between 18 and 30 years old and 56% of the oldest group admitted contact with other people by mobility via public transport, decreasing in the segment between 31 and 45 years old (38.3%). Thus, all the relationships between the respondent’s age and their level of exposure to the virus are significant (*p*-value < 0.05).

### 5.9. Education

The literature announces that personal knowledge about the virus generates an increase in risk perception. On the other hand, the survey showed that of the people who declared having any knowledge about COVID-19, only 67% indicated they had a serious concern about the contagion. One hypothesis to explain the difference between the survey results and the literature is that when there is more information and knowledge about the disease, protection methods increase. In the survey, 87% of people who claimed to know about the disease indicated that they always took self-protection measures, so their concern was minor.

### 5.10. Self-Protection Measures

**Figure 8:**
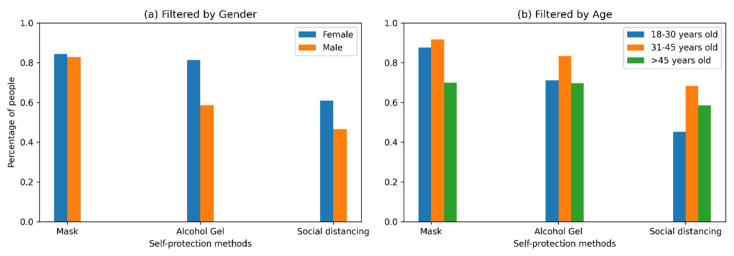
Self-protection methods by gender and age.

For the analysis, we considered that to avoid contagion, people always used self-protection measures, which is why the original categories presented in Appendix A of the survey have been reduced to a binary coding: (1) if the person declared they always used self-protection measures and (2) if not. In this context, self-protection measures such as masks (84%), alcohol gel (74.2%), and social distancing (56.4%) were much more used than gloves (6%) and face shields (4.3%). In addition, in Figure 8 considerable differences were observed between behavior by gender, where 81.3% of female respondents stated that they always used alcohol gel, whereas only 58.6% of male respondents said they did so. We observed similar behavior in social distancing, where 60.9% of women always used this self-protection measure in contrast to 46.6% of men. Regarding the use of masks, there were no significant differences between genders.

An analysis based on age segments indicates that the use of masks presented significant differences (*p*-value < 0.05): 91.7% of people in the range 31–45 years always used masks, a similar percentage to that of people between 18 and 30 years (87.7%). However, a decrease was observed in the older population (45 years and older), in which only 69.8% used a mask continuously. In the case of the use of alcohol gel, 83.3% of the segment 31–45 years old declared that they always used this self-protection measure, in contrast to 71.2% of people 18–30 years old and 69.7% for people from the oldest segment. Regarding social distancing, the segment between 31 and 45 years of age continues to be the most concerned with taking self-protection measures (68.3%), in comparison with the oldest group (58.5%) and the youngest group (45.2%).

## 6. Discussion

The factors identified under the risk homeostasis approach and Lindell and Perry’s protection model are discussed in the following paragraphs:

### 6.1. Gender

The surveys conducted worldwide identified a difference in the perceived risk of COVID-19 among women and men. Women showed a higher risk perception than men. Some authors also declare that women tend to feel more vulnerable in incidents that affect their health, which means that they promptly adopt preventive measures, such as social distancing [13]. However, men are reluctant to adopt protection measures rapidly when facing high severity diseases [7,12].

This difference between individuals’ perceptions could be due to the fact that the severity of disease and number of deaths from COVID-19 are higher among women than men [12]. This perception is a subjective psychological construct influenced by cognitive, emotional, social, and cultural differences [9].

### 6.2. Age

Some researchers indicate that people still do not believe in the risks associated with COVID-19, especially young adults [7]. In several countries, they are the ones who are less concerned about adopting measures of protection. This minor concern could arise from inaccurate information delivered by different media, such as social networks, which are more used by this age range. This low-risk perception is prevalent in this age range, which has the lowest mortality range, since most of the people show less aggressive symptoms and some of them show no symptoms at all as they are asymptomatic ones. Due to this low probability, people in this group of young adults do not adopt self-care measures promoted by the government to prevent contagion [20]. One effect of this misinformation is that 80% of infected people by COVID-19 are between 20 and 59 years old.

Another group affected is older adults who have been most affected by confinement due to quarantine. Research has highlighted the psychological impacts of confinement, quarantine, and isolation due to COVID-19, an impact that can be long-lasting and severe for some cases. In these cases, older adults have the fewest options to get out of confinement and face significant problems in the psycho-emotional sphere [12].

### 6.3. Socioeconomic Level

According to a survey for the BBC, the pandemic is widening the gap between rich and emerging countries. In the survey, 69% of people in emerging countries reported a loss of income, compared to 45% of people in wealthier countries [20]. This loss of income underlines the importance of being more concerned about the virus in low-income households because they must deal with economic tightness and the pandemic effects simultaneously. This puts them in a riskier situation that could make them even more vulnerable because of overcrowding, poor nutrition, and the more significant number of infections in their area [1].

When we talk about people’s ability to recover from any hazard, we can distinguish three cases. First, people with high socioeconomic income have a low vulnerability due to their economic capacity to cope with illness and achieve rapid income recovery. Second, there are people in the middle class who have a lower level of recovery but at the same time have more formal jobs to cope with different events. Finally, the groups with more significant economic needs or low income do not have a great capacity to recover economically in the face of an adverse event for society [21].

The economic impact of the COVID-19 epidemic on Chilean households was job losses and substantial drops in income [22]. According to the results of a survey conducted by Techo para-Chile, households living in camps or with lower incomes have significant disadvantages in facing the COVID-19 epidemic due to the existence of several factors that differ from those with a better economic position, such as a large number of informal jobs, insufficient sanitary infrastructure, urbanization deficit, and lack of connectivity. In addition, the risk perception of these people is high and increases depending on their age, which implies their distrust in finding, for example, an intensive care bed in case they need it [23].

### 6.4. Education Level

According to research, people who have more information related to COVID-19 have a higher perception of risk due to their knowledge about the topic, be it the pros or cons of the situation [9].

Research reports that the social confidence of hazard managers is strongly correlated with judgments about the risks and benefits of the hazard. For example, Siegrist and Cvetkovich [19] conclude in their research that when a person lacks or has no knowledge about a hazard, the social trust of the authorities managing the hazard determines the perceived risks and benefits. In contrast, if a person has personal knowledge about a hazard, he does not need to rely on the managing authorities, so his social trust is not related to the judged risks and benefits. Consequently, the education level of an individual on a specific subject, such as a new disease, will affect criteria when deciding to prevent it and how each person perceives the risk.

### 6.5. Media

Currently, there is a variety of sources of information to obtain data. The choice of these is conditioned by the different personal interests, time available, and age of individuals. According to Salas [7], the lack of perception may be due to the scarce or inadequate information present in the new technologies that children and young people access today. Based on the answers to a survey conducted by Kwok et al. [13] and other researchers from Hong Kong, the most reliable sources of information for the search of data regarding COVID-19 are medical publications, radios, and newspapers [13]. Newspapers and television provided most of the information throughout the pandemic, where television was preferred by 47% of people, and closely followed by printed newspapers with 30%.

This information consumption ratio is why young people tend to have a lower risk perception due to less reliable communication channels, such as Twitter and Facebook. In addition, a study conducted by Muñiz and Corduneanu [24] reveals an important influence of the communicative variables in the generation of judgments about the personal consequences of the pandemic. In this form, the media impacts the perceived risk due to the information transmitted concerning the crisis.

The risk perception approach establishes that the spread of diseases creates psycho-emotional effects on people. However, it also warns of the emergence of distrust towards the government, the media, and especially public health institutions with their forms of communication and the management of contagions, isolation, and mitigation of cases. Consequently, it is crucial to consider that the media and social networks influence people’s perception of risk [2].

### 6.6. Closeness to COVID-19 Cases

The risk perception model recommends incorporating groups of variables corresponding to the cognitive tradition, the emotional and experiential tradition, the sociocultural paradigm, and relevant individual differences. For example, the emotional and experiential tradition allows us to identify, based on surveys conducted in several countries around the world, that people who have a direct personal experience with COVID-19 tend to perceive greater risk compared to those who have not experienced the immediate impact of COVID on their relatives or friends. People who have received information about the disease from people close to them, whom they trust, likewise perceive more risk [9].

Evidence of this perception factor is the example of the study conducted on SARS. This study shows that those who communicated in some way with infected people felt more fear, anger, sadness, and guilt because of the possibility of getting the disease [8].

More specifically, many analyses show that people’s perception of risk is higher when people have direct personal experience with the virus, and there is also talk that there is equally higher risk perception among those who have more pro-social worldviews [9].

## 7. Limitations of the Study and Further Research

One of the limitations of this research is the type of sample and the sample size considered for the survey analysis, since it does not manage to represent all the variability of the population in Chile, as it was an exploratory survey. Likewise, at the time of the survey, there was no information available at the national level to compare different scenarios, and the lockdowns made meetings and field trips challenging to collect more information. Therefore, it is suggested to calculate an appropriate sample size for Chile and to conduct a survey with a significant coverage that would allow the generation of more detailed public policies of more significant impact in the community.

We suggest investigating further the relationship between the acceptance of protection methods and the proximity and experiences of individuals with COVID-19. Additionally, the relationship between the same methods and the difference in people’s perception according to the community or sector where they live is essential due to the overcrowding in some communities with high population density.

Furthermore, risk perception modeling requires granularity regarding personal decision-making for COVID-19 prevention measures’ adoption. Further studies must weigh the relevance of geographic locations and sociocultural identification. These factors should be able to identify patterns and avoid biases from noise. Even though variability exists, later studies should pay attention to complex pattern identification to forecast and estimate factors of judgement for decision-making.

## 8. Conclusions

This research contributes to identifying the main factors that influence how individuals face risk and how they have faced COVID-19. The proposal of risk homeostasis, where there are aspects that help to form an accepted level of risk and aspects that help to form a perception of risk, allowed identifying intrinsic aspects of individuals that model the level of risk they are willing to tolerate, and the environment provides them with information that allows them to generate a perception of the risk to which they are exposed. In addition, the risk homeostasis approach helps to understand that individuals generate different responses; some may take protective measures, and others may not take measures to protect themselves from the risk of contagion. This variation in the actions taken by an individual can be explained on the basis that individuals have a different appreciation of the same risk.

The Lindell and Perry model was applied to model the decision-making process for implementing protective actions. This model made it possible to model the factors that affect decision-making for protective actions and, although this model was designed mainly to explain socio-natural disasters that usually affect the population in shorter periods, unlike the pandemic, it made it possible to understand the different influences that factors have on the adoption of protective measures by the population. Specifically, facilitators and situational impediments are increasingly important in adopting protective measures that are transformed into long-term life habits—likewise, the importance of the psychological aspect in adopting protective actions increases. Therefore, in this case of a pandemic, situational facilitators and situational impediments must be considered in public policies, companies, and society to reduce the damage to mental health caused by long periods away from loved ones or the stress caused by the lack of resources due to job losses. This damage to mental health becomes more critical when a percentage of the population performs activities outside the formal economy, for which the state has no records and no mechanisms to support individuals when they reduce their income or when businesses or SMEs are associated with gastronomic or hotel services that the lockdown has strongly impacted.

The literature review allowed us to identify the aspects that have the most significant impact on accepted risk; for example, gender, age, socioeconomic level, and educational level. On the other hand, the aspects that seem to have the most significant influence on the perception of risk appear to be the means of information used and the proximity to cases of COVID-19. In the exploratory survey, the factor of gender is one factor where there was the most significant difference in the perception of risk and the decision to accept the protective measures. Another factor is the age of individuals, who show a difference in their perception of risk based on the different age ranges. Thirdly, education and socioeconomic level generate differences in the adoption of protective measures because the levels of concern about the disease are different among individuals with different levels of education. Finally, it is essential to highlight that even though there is a high level of distrust in the authorities, most citizens adopt at least one of the measures indicated by the authorities.

Identifying segments of the population with less concern about contagion makes it possible to focus efforts to educate and raise awareness of the effects of the disease and promote healthier habits for society in these segments. In Chile, efforts need to be directed to men and young people to promote more measures of protection, primarily a focus on social distancing. Additionally, among the factors mentioned in the research, emphasis should be given to programs that promote mental health issues present in the population, and according to the survey conducted, the percentage of the population affected exceeds 50%.

## Data Availability

Data available on request due to privacy/ethical restrictions.

## Appendix A. COVID-19 Risk Perception Survey

This study’s objective is to support conclusions providing information related to the characteristics of individuals in Chile, and with this to understand if there are differences in risk perceptions and decision-making when faced with COVID-19.

Please read each of the questions carefully and answer according to your personal characteristics and appreciation, considering that your personal data will be processed only for the purposes of this research. It will take between 4 to 6 min to answer the survey, and you will have the option to withdraw from this survey at any time you wish.

Click on the alternative of your choice.

**Section 1: Personal Background**

1. Gender.

___ Female
___ Male
___ Prefer to not answer

2. Age.

___ 18–30 years old
___ 31–45 years old
___ 46–60 years old
___ More than 60 years old

3. Residence Town

______________________________________

4. Housing.

___ House
___ Apartment
___ Other

5. Number of people you live with in your household.

___ 1–3 persons
___ 4–6 persons
___ More than 7

**Section 2: Perception assumed by each interviewee.**

6. Have you had symptoms related to COVID-19 during the pandemic (fever, dry cough, tiredness, nasal congestion, headache, loss of taste or smell)?

___ YES
___ NO

7. Have you ever been tested for COVID-19?

___ YES
___ NO

8. Do you have, or have you ever had COVID-19?

___ YES
___ NO

9. How concerned are you about getting COVID-19?

___ I am very concerned.
___ I am concerned.
___ I am a little worried.
___ I am not worried.

10. Regarding the previous question, why are you concerned? Check all the necessary options.

___ Because you must attend work.
___ Because you use public transportation.
___ Because you go to buy supplies.
___ Because you risk infecting family members.
___ Because you have underlying illnesses that may present with severe illness due to COVID-19.
___ Because you take all precautionary measures.
___ Because you do not believe that COVID-19 exists.

11. Does anyone close to you have or have they had COVID-19?

___ YES
___ NO

12. How concerned are you that someone close to you might get COVID-19?

___ I am very concerned.
___ I am worried.
___ I am a little worried.
___ I am not worried.

13. Regarding the previous question, why are you concerned? Please check all that apply.

___ Because you must go to work.
___ Because you use public transportation.
___ Because you go to buy supplies.
___ Because you have underlying illnesses that may present with severe disease due to COVID-19.
___ Because you risk infecting family members.
___ Because my family does not believe that COVID-19 exists.

14. How often have you felt depressed, afraid, or anxious because of COVID-19?

___ Never, since the pandemic began.
___ Once every three months.
___ Once a month.
___ Once a week.
___ Always, since the pandemic began.

**Section 3: Factors that may influence the change of perception**.

15. In terms of the information provided by the mass media to inform you about COVID-19, do you trust this information?

___ YES
___ NO

16. Which mass media do you use regularly to be updated on COVID-19 developments? Check more than one option if necessary.

___ Radio
___ Television
___ Social media
___ Printed newspapers
___ Other ______________________

17. Do you trust the information provided by the country’s authorities?

___ Yes, I trust.
___ Yes, I trust a little.
___ No, I do not trust.

18. Do you have any degree of knowledge about how COVID-19 behaves and what it is?

___ YES
___ No
___ Somewhat

19. Have you participated in the last week in a group meeting with people outside of your immediate family?

___ Never.
___ Between 1 to 3 times.
___ Between 4 to 6 times.
___ More than 7 times.

20. In the last month, have you done any of the following?

Go to work outside the place where you are currently living. ___ Yes ___ No
Go to a restaurant or shopping mall. ___ Yes ___ No
Go to a supermarket, pharmacy, or farm market. ___ Yes ___ No
Have you used public transportation? ___ Yes ___ No
Spent time with people who do not currently live with you? ___ Yes ___ No
Attended a public event with more than the allowed number of people? ___ Yes ___ No

21. How often do you use any of the COVID-19 protection methods when leaving your home?

___ Never.
___ Sometimes.
___ Most of the time.
___ Always.

22. Check all the COVID-19 protection methods you have used below.

Mask ___ Never ___ Sometimes ___ Almost always ___ Always
Distancing ___ Never ___ Sometimes ___ Almost always ___ Always
Wash your hands with soap or sanitized ___ Never ___ Sometimes ___ Almost always ___ Always
Ware Gloves ___ Never ___ Sometimes ___ Almost always ___ Always
Ware Face Mask ___ Never ___ Sometimes ___ Almost always ___ Always

23. Has your household income changed during the pandemic?

___ Your income decreased.
___ Your income has remained the same.
___ Your income increased.
___ Don’t know the variation.

24. Do you have access to soap and water for good hygiene where you live now?

___ YES
___ No

25. Level of education (indicate up to which level of education you have completed).

___ Elementary School
___ High School
___ University
___ Other

26. Average household income per inhabitant before the pandemic.

___ Between CL$ 20,000 and CL$ 63,000.
___ Between CL$ 63,001 and CL$ 107,000.
___ Between CL$ 107,001 and CL$ 169,000.
___ Between CL$ 169,001 and CL$ 302,000.
___ More than CL$302,000.
